# Nuclear Wiskott–Aldrich syndrome protein co-regulates T cell factor 1-mediated transcription in T cells

**DOI:** 10.1186/s13073-017-0481-6

**Published:** 2017-10-27

**Authors:** Nikolai V. Kuznetsov, Bader Almuzzaini, Joanna S. Kritikou, Marisa A. P. Baptista, Mariana M. S. Oliveira, Marton Keszei, Scott B. Snapper, Piergiorgio Percipalle, Lisa S. Westerberg

**Affiliations:** 1 Department of Microbiology Tumor and Cell biology, Karolinska Institutet, Stockholm, 171 77 Sweden; 2 Department of Cell and Molecular Biology, Karolinska Institutet, Stockholm, 171 77 Sweden; 3 King Abdullah International Medical Research Center/King Saud bin Abdulaziz University for Health Sciences Medical Genomic Research Department, MNGHA, Riyadh, Saudi Arabia; 4 Institute for Virology and Immunobiology, University of Würzburg, 97078 Würzburg, Germany; 5 Gastroenterology Division, Children’s Hospital, Harvard Medical School, Boston, MA 02115 USA; 6 Biology Program, New York University Abu Dhabi (NYUAD), P.O. Box 129188, Abu Dhabi, United Arab Emirates; 7 Department of Molecular Biosciences, The Wenner-Gren Institute, Stockholm University, 106 91 Stockholm, Sweden

**Keywords:** WASp, T cells, ChIP-seq, Wiskott–Aldrich syndrome, TCF1, TCF12, Nucleus

## Abstract

**Background:**

The Wiskott–Aldrich syndrome protein (WASp) family of actin-nucleating factors are present in the cytoplasm and in the nucleus. The role of nuclear WASp for T cell development remains incompletely defined.

**Methods:**

We performed WASp chromatin immunoprecipitation and deep sequencing (ChIP-seq) in thymocytes and spleen CD4^+^ T cells.

**Results:**

WASp was enriched at genic and intergenic regions and associated with the transcription start sites of protein-coding genes. Thymocytes and spleen CD4^+^ T cells showed 15 common WASp-interacting genes, including the gene encoding T cell factor (TCF)12. WASp KO thymocytes had reduced nuclear TCF12 whereas thymocytes expressing constitutively active WASp^L272P^ and WASp^I296T^ had increased nuclear TCF12, suggesting that regulated WASp activity controlled nuclear TCF12. We identify a putative DNA element enriched in WASp ChIP-seq samples identical to a TCF1-binding site and we show that WASp directly interacted with TCF1 in the nucleus.

**Conclusions:**

These data place nuclear WASp in proximity with TCF1 and TCF12, essential factors for T cell development.

**Electronic supplementary material:**

The online version of this article (doi:10.1186/s13073-017-0481-6) contains supplementary material, which is available to authorized users.

## Background

The actin cytoskeleton is essential for life and regulates critical cellular functions including morphogenesis, migration, cytokinesis, membrane transport, and cell-to-cell communication.

In addition to its fundamental role in the cytoplasm, actin resides in the nucleus and regulates chromatin remodeling, RNA processing, and nucleoskeletal stability and transcription [1]. Nuclear actin serves multiple functions, both dependent and independent, on its role in polymerization of actin filaments [2–5]. Spontaneous actin assembly is inefficient and the cell uses a number of actin nucleating promoting factors to obtain rapid and dynamic actin polymerization [6]. The Wiskott–Aldrich syndrome (WASp) family of nucleating promoting factors all contain a verprolin-cofilin-acidic (VCA) domain that binds to the actin-related protein (Arp)2/3 complex to induce actin polymerization leading to formation of branched filaments [6]. It has become increasingly clear that actin nucleating promoting factors such as the WASp family are present both in the cytoplasm and in the nucleus. The WASp family of proteins includes WASp, neuronal (N)-WASp, and WASp-family verprolin-homologous protein (WAVE)/suppressor of the cyclic AMP receptor (SCAR) 1-3, WASp and SCAR homologue (WASH), and junction-mediating and regulatory protein (JMY) [7]. The activity of WASp, N-WASp, and WAVE/SCAR 1-3 are regulated by the Rho family GTPases Cdc42, Rac1, and Rac2. At rest, WASp and N-WASp resides in an auto-inhibited conformation due to an intramolecular interaction between the VCA domain and the GTPase-binding domain. Upon binding of Cdc42, the auto-inhibited conformation is released and exposes the VCA domain that allows for recruitment of the Arp2/3 complex and actin polymerization [8–10]. Rac1 and Rac2 regulate activation of the multimeric WAVE/Scar regulatory complex to stimulate actin polymerization by the VCA domain [11–13].

All steps of T cell development in thymus is coordinated by the actin cytoskeleton to control cycles of T cell migration and stable immunological synapse assembly with antigen-presenting stroma cells and dendritic cells [14]. Moreover, the development of T cells from multipotent progenitor cells depends on fine-tuned regulation of transcription factors (TFs), resulting in the loss of alternative lineage potential and the acquisition of the T cell functional identity. This lineage commitment relies on Notch signaling and the activity of TFs such as the high mobility group (HMG) family of TFs T cell factor (TCF)1, lymphoid enhancer factor (Lef)1, and the E-box proteins TCF12/HeLa E-box-binding protein (HEB), E2-2, and E2A [15, 16]. In thymus, T cells develop from CD4^–^CD8^–^ double-negative thymocytes to express a functional T cell receptor (TCR) upon which they become CD4^+^CD8^+^ double-positive thymocytes. The CD4^+^CD8^+^ double-positive thymocytes constitute more than 80% of all cells in the thymus and develop into CD4^+^CD8^–^ and CD4^–^CD8^+^ single-positive T cells that leaves the thymus and enter into the circulation as mature CD4^+^ and CD8^+^ T cells.

WASp is uniquely expressed in hematopoietic cells and its role for their normal function have been extensively studied in Wiskott–Aldrich syndrome (WAS) patients that lack expression of WASp and in gene-targeted mice (WASp KO mice) [17, 18]. These studies have revealed WASp to be critical for correct T cell function and 40–70% of WAS patients develop T cell-mediated autoimmunity [17–22]. This could at least in part be due to a limited and more autoreactive T cell receptor repertoire in peripheral blood T cells from WAS patients [23–26] and in thymocytes and spleen T cells from WASp KO mice [27]. WASp KO thymocytes and T cells show decreased proliferation in response to T cell receptor stimulation [21, 28, 29] and this is associated with reduced translocation of nuclear factor of activated T cells (Nfatc)2 into the nucleus of WASp KO T cells upon T cell receptor stimulation, leading to decreased expression of interleukin-2 [30–32]. X-linked neutropenia (XLN) is a more recently described WASp-associated disease caused by point mutations (WASp^L270P^, WASp^S272P^, WASp^I276S^, and WASp^I294T^) in the WASp GTPase-binding domain that renders WASp constitutively active by destroying the autoinhibited folding of WASp [33–37]. Murine spleen T cells expressing WASp^L272P^ and WASp^I296T^ (corresponding to human WASp^L270P^ and WASp^I294T^) have an increased load of polymerized actin associated with decreased cell spreading [37].

Using a chromatin immunoprecipitation (ChIP) on array approach including 50 genes known to regulate T helper (Th)1 and Th2 commitment, Vyas et al. showed that nuclear WASp in peripheral blood T cells interacts specifically with Tbx12-induced genes for Th1 commitment [38]. Moreover, using in vitro derived Th1 cells, WASp interacts with a subset of SWItch/Sucrose Non-Fermentable (SWI/SNF) complexes to drive transcription of Tbx12-induced genes [39]. Other interaction partners of WASp in the nucleus remain to be identified. Although WASp has been extensively investigated in proximity to T cell receptor signaling in the cytoplasm, the role of nuclear WASp for T cell development remains to be identified and should be important to fully understand the pathophysiology of WASp-associated immunodeficiency diseases.

To identify the global gene occupancy by WASp in the nucleus during T cell development, we took the unbiased approach to perform ChIP sequencing (ChIP-seq) and compare thymocytes (immature T cells) and spleen CD4^+^ T cells (mature T cells). We identify 15 common WASp-enriched genes, including the gene encoding for TCF12. By comparing wild type (WT), WASp KO, WASp^L272P^, and WASp^I296T^ thymocytes from gene-targeted mice, our data show that the activation status of WASp affects TCF12 protein in the nucleus. We identify a putative WASp DNA-binding site identical to the TCF1-binding site and we show that WASp interacted with TCF1 in the nucleus. Together, we define that nuclear WASp acts in proximity with TCF1 and TCF12, important for normal T cell development.

## Methods

### Mice

WASp^L272P^ mice (C57Bl/6 ES cells, C57Bl/6 background) and WASp^I296T^ mice (mixed 129Sv and C57Bl/6 ES cells, backcrossed for eight generations to C57Bl/6 background) were generated by insertion of a point mutation into germline *WASp* exon 9; TTG to CCG to generate the Leu-272 to Pro-272 knock-in mutation or ATT to ACT to generate the Iso-296 to Thr-296. The WASp^L272P^ and WASp^I296T^ knock in strains were generated by Ingenious Targeting Laboratory. WASp KO (C57Bl/6 background) [40], WASp^L272P^, WASp^I296T^, and littermate age- and sex-matched WT mice were bred and maintained at the animal facility of the Department of Microbiology, Tumor and Cell Biology at Karolinska Institutet under specific pathogen-free conditions. Males were used at six weeks of age and all animal experiments were performed after approval from the local ethical committee (the north Stockholm district court); protocol approval number: N77/13.

### Mouse primary cells and cell cultures

WT, WASp KO, WASp^L272P^, and WASp^I296T^ thymocytes were isolated by thymi homogenization through 100-μm strainers (Corning) and cell resuspension in sterile cold 1x PBS. Ten thymi of a specific genotype were pooled and used per ChIP experiment. WT, WASp KO, WASp^L272P^, and WASp^I296T^ CD4^+^ T cells were isolated by spleen homogenization through 100 μm strainers (Corning) followed by splenocytes-derived T cell isolation using CD4^+^ T Cell Isolation Kit (Miltenyi). The purity of CD4^+^CD3^+^ T cells was 94–96% (Additional file 1: Figure S1). Cell lines used: murine fibroblast L-cells (ATCC no. CRL-2648) and A20 (mouse reticulum cell sarcoma lymphoblast lymphoblastoma) cell line (ATCC no. TIB-208). Experiments that were from different biological samples (cells or mice) and performed at least twice and consistently measured are considered biological replicates. Consistent measurements of one biological sample performed at least twice are considered technical replicates.

### Cell fractionation and immunoprecipitation (IP)

Mouse thymi were collected and cell suspension prepared with the use of 100-μm strainers (Corning). For cell fractionation, the Nuclear/Cytosol Fractionation Kit #K266-25 (BioVision) was used. For IP, a protease inhibitors cocktail (SigmaAldrich) was added to all buffers and lysis solutions. 10^8^ cells/mL were washed twice with cold 1x PBS containing a protease inhibitors cocktail (Sigma-Aldrich), sedimented in Eppendorf centrifuge (2000 rpm, 5 min, 4 °C). Total cell lysates were prepared by incubation on ice for 30 min of the cell pellet in equal volume of lysis buffer containing NP-40, followed by centrifugation (14,000 rpm, 15 min, 4 °C). Supernatants were collected and pre-cleared by adding 100 μL of pre-equilibrated rec-Protein G-Sepharose® 4B Conjugate (Invitrogen) suspension per sample. Ten to 20 μL of antibodies were added to pre-cleared supernatant and immunoprecipitation performed during 10 h at 4 °C on shaking platform. Afterwards, 100 μL of pre-equilibrated protein G-Sepharose beads suspension per sample was added to the IP samples with continued incubation during 4 h at 4 °C on shaking platform. Triple wash of IP samples was performed via beads re-suspension in 1 mL of cold 1x PBS followed by centrifugation (1000 rpm, 3 min, at 4 °C) and collection of supernatant. One hundred microliter aliquots of loading and three washes were collected and stored frozen at –20 °C. Bead pellets were re-suspended in 300 μL of 4x Laemmli gel loading buffer. Samples were heated at 95 °C in a solid block thermostat for 10 min and centrifuged (1500 rpm, 5 min, room temperature). Collected supernatant aliquots were stored at –20 °C and used for western blotting analysis as described. Ten to 20 μL of purified mouse IgG_2A_ (#401501, Biolegend) was used as the isotype antibody (Ab)-negative control in co-IP experiment.

### Chromatin immunoprecipitation (ChIP)

**Fig. 1:**
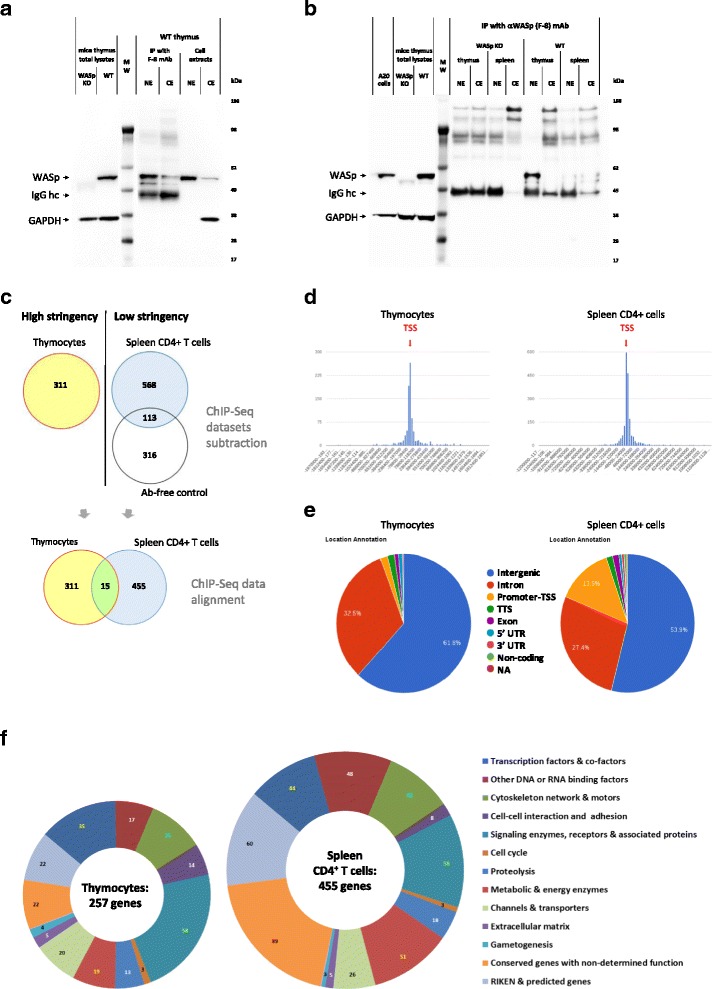
ChIP-seq analysis of WASp enriched genes. **a** The F-8 anti-WASp Ab specifically recognizes WASp in total thymocyte lysates, nuclear extracts (NE), and cytosolic extracts (CE). Heavy chain of mouse IgG (IgGhc) is observed in eluted IP samples. GAPDH was used as a sample loading control for total lysates. **b** WASp is abundant in NE of WT thymocyte when compared to NE of WT spleen CD4^+^ T cells. Heavy chain of mouse IgG (IgGhc) is observed in eluted IP samples. GAPDH was used as a sample loading control for total lysates. These data are representative of four (thymus fractionation) and two (spleen CD4^+^ T cell fractionation) independent biological replicates. **c** ChIP-seq analysis of thymocytes and spleen CD4^+^ T cells flowchart. WASp-enriched genes by ChIP-seq analysis of WT thymocytes performed under high stringency condition. WASp-enriched genes by ChIP-seq analysis of spleen CD4^+^ T cells performed under low stringency conditions and compared to a ChIP-seq dataset from control Ab-free spleen CD4^+^ T cells. All common genes were subtracted from the spleen CD4^+^ T cells dataset. Both ChIP-seq datasets were then aligned and compared to identify common genes in thymocytes and spleen CD4^+^ T cells. **d** Distribution of distance from WASp ChIP-seq gene peaks to the nearest transcription start site (TSS) defined according to the positions of RefSeq TSSs using the CHIPseek software. Negative values indicate 5’ of the TSS, positive values indicate 3’ of the TSS. **e** Annotation of WASp ChIP-seq gene peak positions within intergenic and genic regions using CHIPseek software. By default promoter-TSS annotation defined as area from – 1 kb to + 100 bp of the TSS position and transcription termination site area (TTS) defined from – 100 bp to + 1 kb of the TTS position. **f** Functional groups of peak-corresponding genes in WASp ChIP-seq refined datasets. Additional file 1: Figure S1–S3 and Additional file 3: sheets 1 and 2

Three rounds of sample preparation for ChIP-seq were made. (1) High stringency condition was used for ChIP of WT thymocytes with the WASp Ab or an isotype control. Only the WASp ChIP sample could be used for sequencing since the isotype control sample had too low DNA concentration by Bioanalyzer analysis for sequencing (Additional file 1: Figure S2B). (2) To obtain enough DNA for sequencing, the WT spleen CD4^+^ T cell sample was prepared under low stringency for WASp ChIP (Additional file 1: Figure S2C). An Ab-free WT spleen-negative control sample was sequenced and all hits present in this sample were subtracted from the WASp ChIP-seq data of the WT spleen CD4^+^ T cells (Fig. 1c). (3) ChIP samples from WT, WASp^I296T^, and WASp KO thymocytes were prepared under low stringency. The WASp KO thymocyte sample had too low DNA concentration by Bioanalyzer for sequencing (Additional file 1: Figure S2D). To detect differential DNA interaction between WASp^I296T^ and WT WASp, we removed all similar hits between the two samples to identify the unique genome occupancy by WASp^I296T^. The ChIP protocol was performed with high stringency or low stringency in regard to fixation and washing conditions. Two milliliters of single-cell suspension per ChIP sample containing 1–2 × 10^8^ cells were used. For high stringency, cells were cross-linked with 1% FA for 15 min, and for low stringency, cells were cross-linked with 10 mM DMA (linker arm length 8.6 Å, Pierce) for 1 h ± 0.75% FA for 5 min. Cross-linking was followed by a 20-min incubation with 137 mM glycine to quench the formaldehyde cross-linking. Cells were washed twice with cold 1x PBS, harvested by centrifugation, resuspended in 1x PBS, and briefly sonicated on ice using Bioruptor UCD-200 sonication system (Diagenode) at a setting of 5 for 30-s pulses with a 30-s pause between pulses. Cell lysates were treated with 100 U/mL benzonase (Merck) for 40 min at room temperature with slow rotation and the nuclease reaction was stopped by addition of EDTA to a final concentration of 5 mM. After quenching of the benzonase reaction, the samples were centrifuged at 20,000 g for 15 min at 4 °C. The supernatant was collected and 10% (volume) of the supernatant was stored (–20 °C) for further ChIP-seq input control DNA analysis. Ten microliters of monoclonal anti-WASp F-8 Ab (Santa Cruz Biotechnology) were added to the rest of pre-cleared supernatant and immunoprecipitation was performed during 10 h at 4 °C on shaking platform. Resulted ChIP samples were added to 100 μL per sample of pre-equilibrated protein G-Sepharose beads suspension with further incubation during 4 h at 4 °C on shaking platform. Triple wash of IP samples was performed via beads re-suspension in 1 mL of cold 1x PBS followed by centrifugation (1000 rpm, 3 min, 4 °C) and collection of supernatant. For high stringency, the beads were washed twice with RIPA buffer, pH 7.6 (50 mM HEPES-KOH pH 7.6, 100 mM NaCl, 1 mM EDTA, 500 mM LiCl, 1% NP-40, 0.7% sodium deoxycholate). For low stringency, the beads were washed twice with cold 1x PBS or TE buffer containing 50 mM NaCl. Finally, beads were re-suspended in elution buffer, pH 8.0 (1% SDS, 50 mM tris-HCl, 10 mM EDTA) and incubated for 2 h at 65 °C followed by incubation for 10 h at 56 °C with 100 μL/mL proteinase K, #19133 (QIAGEN). Quality control of ChIP DNA fragments was performed using Bioanalyzer 2100 with High Sensitivity DNA Kit, #5067-4626 (Agilent) to ensure that the size of DNA fragments was in the range of 100–800 bp (Additional file 1: Figure S2A). Library construction for ChIP-seq and DNA sequencing (10 M clean data) using the Illumina Genome Analyzer platform was performed at Beijing Genome Institute (BGI, Shenzhen). ChIP validation polymerase chain reaction (PCR) was performed on a number of identified gene targets to confirm specific DNA enrichment vs. ChIP Ab-free negative control sample.

### Western blotting

Western blotting was performed using the Bolt electrophoresis system (Invitrogen) and Bolt™ 4–12% Bis-Tris Plus polyacrylamide gels NW04120BOX (Thermo Fisher Scientific) in 1x Bolt® MOPS SDS Running buffer B0001 (Thermo Fisher Scientific). Blots were processed in 1x TBS-T buffer containing 5% non-fat dry milk #1706404 (Bio-Rad) with antibodies used as follows: WASp F-8 (Santa Cruz Biotechnology), TCF1 H-118 (Santa Cruz Biotechnology) diluted 1:200, TCF12 PA5-21039 (Invitrogen) diluted 1:300, GAPDH (FL-355, Santa Cruz Biotechnology) diluted 1:1000, histone H3 (ab1791, Abcam) diluted 1:10,000. Detection of GAPDH was used as a sample loading control for total lysate and for cytosolic extract. Detection of histone H3 was used as a sample loading control for nuclear extract. Blots were developed using IgG-HRP labeled antibodies (Santa Cruz Biotechnology) and the ECL detection system. Blot images were analyzed using the ImageQuant LAS 4000 LAS4000 Image system using ImageQuant LAS 4000 Control Software (GE Healthcare). The images of pre-stained Protein Standard SeeBlue Plus2 LC5925 (Thermo Fisher Scientific) and sample loading controls derived from the same gel run, were overlaid onto blot image using Adobe Photoshop CS6. The quantification of protein band intensity on western blots was performed using ImageJ software and normalized integrated signal density was determined with Excel software.

### Fluorine-conjugated UTP (FUrd) incorporation transcription assay

For the in vivo run-on assays to monitor nascent precursor RNA polymerase II transcripts [41], the thymocyte cell suspension was incubated with 0.04 μg/mL of actinomycin D in RPMI media for 2 h, then cells were treated with 2 mM FUrd (Sigma-Aldrich) for 15 min. To stop the reaction, cells were washed twice with cold 1x PBS; then the cell suspension was applied on glass slides. Cells were fixed using 3.7% formaldehyde in 1x PBS, permeabilized with 0.5% Triton -X100 in 1x PBS, then incubated with anti-BrdU Ab (Life Technology) to detect FUrd incorporation into nascent RNA. In addition, anti-histone H3 ab1791 (Abcam) was applied for the detection of nuclei pattern. For the microscopy analysis, cells were mounted in Prolong Gold mounting medium containing DAPI (Molecular Probes) and a single image representing the focal plane was captured using Leica DMRXA microscope at × 200 and × 1000 magnifications. Several randomly distributed cells were captured for each sample. Images were processed using Openlab 3.1.4 software (Improvision) and Adobe Photoshop 8.0. For quantification, the cells as defined by DAPI signal were outlined and the integrated density of FUrd fluorescence signal was calculated using ImageJ software. Ten cells with the brightest FUrd signal found on each image were taken for subsequent analysis. The obtained integrated density of the signal was normalized for the main value found in the WT cells. Statistical analysis was performed in GraphPad Prism and Excel software. Chosen sample size covered all available detected signals in the limited dataset of WASp KO mice in the first experimental round. A similar sample size was chosen in available WASp^I296T^ mice in the second experimental round. To create a balanced design, the same sample size was chosen in WT mice in both rounds of experiments. Statistical analysis of nested ANOVA was applied in both rounds of experiments. Analysis in the first experimental round—FUrd signal measurements in WASp KO vs. WT mice—was performed with the following parameters: exact values of N: 90 for each mice (nine images with ten measured values); 180 for each genotype; definition of the center in WT1-3: 9603; 8069; 7686; definition of the center in WASp KO1-3: 3613; 3832; 5150; mean ± SD in WT1-3: 11,420 ± 7007; 9644 ± 5532; 10,019 ± 6145; mean ± SD in WASp KO1-3: 6374 ± 5540; 5448 ± 3959; 7046 ± 4882. Analysis in the second experimental round—FUrd signal measurements in WASp^I296T^ vs. WT mice—was performed with following parameters: exact values of N: 60 for each mice (six images with ten measured values); 240 for each genotype; definition of the center in WT1-4: 782,452; 507,618; 703,523; 577,830; definition of the center in WASp^I296T^1-4: 660,231; 503,051; 517,926; 587,749; mean ± SD in WT1-4: 857,222 ± 434,228; 686,248 ± 518,108; 781,260 ± 452,743; 645,974 ± 299,508; mean ± SD in WASp^I296T^1-4: 753,605 ± 401,440; 564,983 ± 247,523; 701,733 ± 537,852; 612,138 ± 279,542. Exact *p* values in FUrd incorporation transcription assay in the first experimental round (WASp KO vs. WT): *p* = 0.0046; in the second experimental round (WASp^I296T^ vs. WT): *p* = 0.2337.

### Real-time quantitative PCR-based gene expression profiling

Gene specific primers designed with Primer Bank software are listed in Additional file 2. Total RNA from thymocytes was isolated with RNeasy kit #74104 (QIAGEN). Genomic DNA nuclease treatment with RNAse-free DNAse kit # 79254 (QIAGEN) was performed during isolation procedure for each prepared total RNA sample. The concentration of isolated total RNA was measured by NanoDrop 2000 (Thermo Scientific). The visual quality control for RNA integrity and purity was performed by 1% UltraPureAgarose, #16500500 (Invitrogen), 1x TAE gel electrophoresis analysis. Gel images were created with ImageQuant LAS 4000 LAS4000 Image system using ImageQuant LAS 4000 Control Software (GE Healthcare). Two micrograms of total RNA per sample was used for each complementary DNA (cDNA) synthesis reaction (with SuperScript™ II Reverse Transcriptase, #18064-014 (Invitrogen). Real-time quantitative PCR (RT-qPCR) reactions were performed using SsoAdvanced™ Universal SYBR® Green Supermix kit, #1725272SP (Bio-Rad) with C1000/CFX96 RT-PCR System (Bio-Rad). Gene expression profiling by RT-qPCR was performed in three technical replicates for each cDNA template sample with Ct variation between triplicates less than cut-off ± 0.25. Gene expression data: the mean of the technical triplicate, ΔCt, RQ value, and standard deviation between RQ of biological triplicates were calculated and analyzed using Excel software.

### Bioinformatics analysis

Standard bioinformatics analysis of ChIP-seq data was performed by BGI (Shenzhen, Guangdong, China) including primary data filtering to remove adaptor sequences, contamination and low-quality reads from raw reads, read alignment, and genome-wide distribution of ChIP-seq reads (Table 1), general classification of all hits, GO function analysis of peak-related genes, sequence analysis using Motif discovery tool (MEME) and Motif Alignment and Search Tool (MAST). Peaks were identified using peak calling approaches MACS (Model-based Analysis for ChIP-Seq) and SICER (spatial clustering approach for the identification of ChIP-enriched regions) [42–44]. Differential binding peaks were obtained by software MAnorm as described [45]. Functional classification of refined data for peak-related genes was performed using NCBI Gene resource (Bethesda, MD, USA). All hit classification diagrams were made with Excel software. Distribution of distances from gene peak reads to the nearest transcription start site (TSS) were defined according to the positions of RefSeq TSSs using ChIPseek software [46]. Genomic annotation of gene peak positions within intergenic and genic regions was received using ChIPseek software. By default, promoter-TSS annotation is defined as the area from – 1 kb to + 100 bp of the TSS position and the transcription termination site area (TTS) is defined from – 100 bp to + 1 kb of the TTS position [46]. DNA-binding site inference for DNA-interacting protein partner of WASp was assessed by applying TRANSFAC® software [47]. Distribution of activating and repressive epigenetic marks and specific TF sites in selected candidate genes resulted from ChIP-seq data was examined using the UCSC Genome Browser tool suite. Intra-intronic positions of certain peak reads and distances from peak reads to nearest exon of genes selected from ChIP-seq data were identified with UCSC Genome Browser tool suite.

**Table 1:** ChIP sample statistics

Source	Cell type	Sample name	Read length	Clean reads	Mapped reads	Mapped rate (%)	Unique mapped reads	Unique mapped rate (%)
WT thymus sample 1	Thymocytes	Sample2	49	10955383	10625818	96.99	8955583	81.75
WT spleen CD4+ T cells sample	Spleen CD4+ T cells	Sample4	49	31269031	30709811	98.21	27032898	86.45
Ab-free WT spleen negative control 1	Spleen CD4+ T cells	Sample5	49	31288824	30704013	98.13	27032749	86.4
WT thymus sample 2	Thymocytes	Sample12	49	9864439	9538121	96.69	8449710	85.66
WASp thymus I296T sample	Thymocytes	Sample13	49	15526660	15110656	97.32	13339372	85.91
WT thymus input control	Thymocytes	Sample2_input	49	12495831	12274173	98.23	10449083	83.62
WT spleen CD4+ T cells input control	Spleen CD4+ T cells	Sample4_input	49	12658673	12522710	98.93	10976914	86.71

## Results

### WASp localizes to the nucleus in thymocytes

To examine the presence of WASp in the nucleus during development of T cells, nuclear and cytosolic extracts were prepared from thymus single-cell suspension consisting of > 98% thymocytes and from spleen CD4^+^ T cells. We reasoned that WASp may not be an easy protein to ChIP for based on its high homology to N-WASp and the fact that WASp may be associated with actin. To avoid cross-reactivity with N-WASp, we tested a panel of anti-WASp antibodies and screened them on cell lysates from WT and WASp KO thymocytes. We identified that the WASp F-8 Ab specifically detected WASp in total lysates from WT thymocytes while no band was detected in WASp KO thymocytes (Fig. 1a). To examine if WASp was present in the nucleus of thymocytes, nuclear and cytosolic extracts were prepared and WASp was detected both in nuclear and cytosolic extracts and could be immunoprecipitated from the nuclear and cytosolic extracts (Fig. 1a). When comparing immunoprecipitated WASp from nuclear and cytosolic extracts from thymus and spleen CD4^+^ T cells, WASp was almost exclusively detected in the thymus nuclear extract; much less WASp was detected in the nuclear extract of spleen CD4^+^ T cells (Fig. 1b). These data show that the WASp F-8 Ab immunoprecipitates WASp from nuclear and cytosolic extracts of thymocytes and to a lesser extent from spleen CD4^+^ T cells.

### WASp interacts with DNA in both intergenic and genic regions

To investigate the potential association of WASp with the mammalian genome, we prepared thymocyte and spleen CD4^+^ T cells lysates and performed ChIP-seq analysis using the WASp F-8 Ab. Since thymocyte nuclear extracts contained abundant WASp, ChIP-seq was performed under high stringency for cross-linking/washing (1% formaldehyde [FA]/RIPA wash, Additional file 1: Figure S2A, B). The total number of reads were normalized and aligned against the mouse genome to identify regions enriched for WASp binding (Additional file 1: Figure S2E). We identified 311 WASp enriched genes in the WASp ChIP dataset from WT thymocytes (Fig. 1c). For spleen CD4^+^ T cells with less WASp in the nucleus, ChIP-seq was performed under low stringency for cross-linking/washing (10 mM dimethyladipimidate [DMA] ± 0.75% FA/PBS wash, Additional file 1: Figure S2C). To circumvent this difference in ChIP sample preparation between thymus and spleen CD4^+^ T cells, we included an Ab-free control for spleen CD4^+^ T cells performed under low stringency (Additional file 1: Figure S1C). For analysis of spleen CD4^+^ T cells, all genes present in the Ab-free dataset were subtracted from the WASp ChIP sample (Fig. 1c). Since 54 of the genes from the Ab-free dataset were found in the thymocyte WASp ChIP-seq dataset, we excluded these 54 genes from the thymocyte dataset (Fig. 1c). Upon extraction of the sequences in the Ab-free control, the ChIP-seq datasets from thymocytes contained 257 WASp-interacting genes (Fig. 1c and Additional file 3: sheet 1) and 455 WASp-interacting genes from spleen CD4^+^ T cells (Fig. 1c and Additional file 3: sheet 2). When the ChIP-seq datasets from thymocytes and spleen CD4^+^ T cells were compared, 15 common WASp-interacting genes were identified (Fig. 1c). To confirm efficiency of performed ChIP procedure, selected genes were validated by RT-qPCR using the ChIP samples from thymocytes (Additional file 1: Figure S2G, H). Moreover, we sequenced the thymocyte and spleen CD4^+^ T cell input ChIP samples and found more than tenfold enrichment of WASp ChIP-seq enriched genes (Additional file 1: Figure S3). In thymocytes and in spleen CD4^+^ T cells, WASp was clustered around the TSSs (Fig. 1d) and WASp interacted with both intergenic and genic regions of which the majority were intron sequences (Fig. 1e). Functional clustering of these genes using the NCBI Gene resource revealed that WASp was associated with RNA Polymerase II genes involved in a wide range of biological function that were divided into 13 functional groups (Fig. 1f). Among identified genes are TFs and co-factors (Nfatc2, TCF12, Mllt1, Rprd1b, Rprd2, Taf1b, Zfp438), other DNA and RNA-interacting proteins (Rad51d, Exosc10, Rbm20), regulators of signal transduction (Arhgap26, Phlpp1, Vav2, Vav3), cytoskeleton-associated proteins (Alms1, Cenpe, Nebl, Palld, WAVE2), and molecular motors (Myh9, Myh10; see Additional file 3: sheets 1 and 2).

### Genome-wide DNA occupancy of WASp associated with RNA Polymerase II genes

**Fig. 2:**
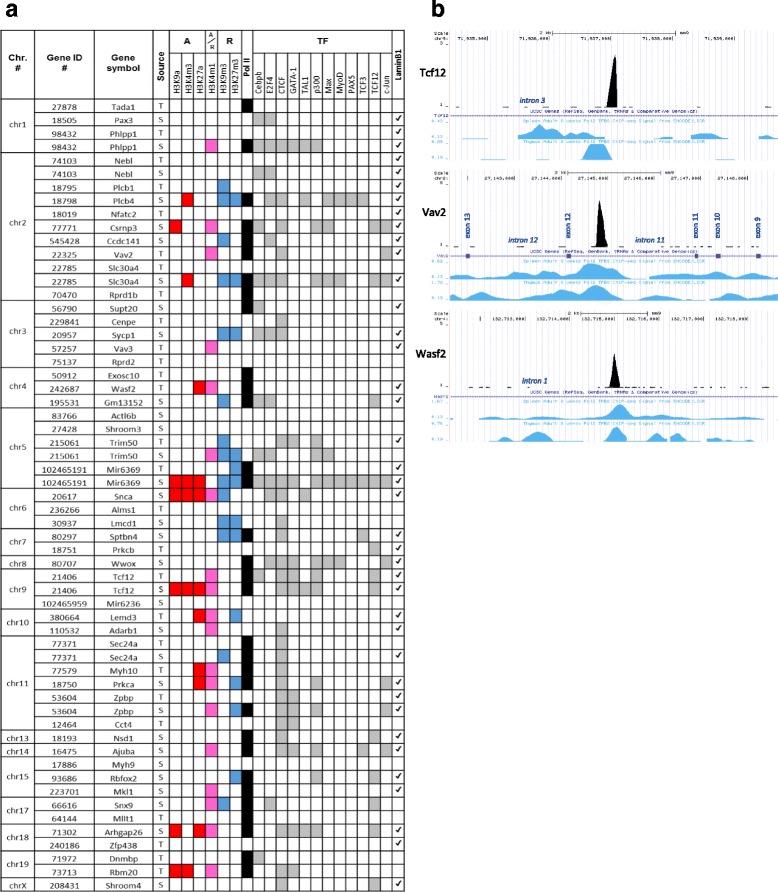
Transcription matrix for selected WASp enriched genes. **a** Bioinformatic association of WASp ChIP-seq peaks from our datasets with activating (A) and/or repressive (R) epigenetic regulatory marks, RNA Polymerase II and TFs peaks and lamin B1 marks in thymocytes (T) and spleen CD4^+^ cells (S). **b** Association of WASp ChIP-seq peaks in thymocytes of *Tcf12*, *Vav2*, and *Wasf2* with RNA Polymerase II ChIP-Seq signal by TFBS ENCODE/LICR resource at UCSC genome browser

**Fig. 3:**
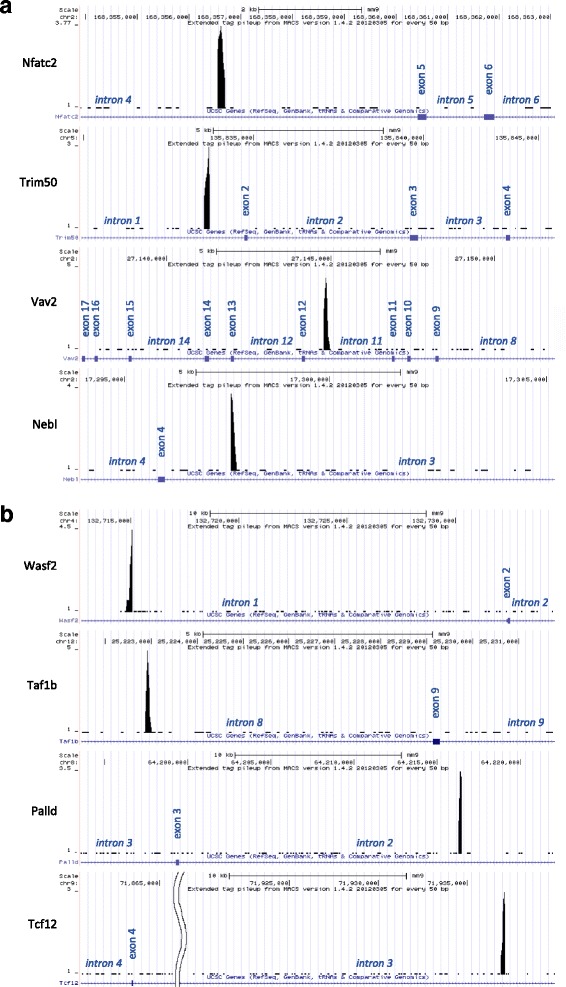
Variation of distances from WASp ChIP-Seq gene peaks to nearest exon(s). Selected examples of genes with (**a**) shorter distance range (0.5–5 kb) and (**b**) longer distance range (5–50 kb) between WASp ChIP-seq gene peak position and nearest exon. Additional file 1: Figure S4

We used the tools available at the University of California Santa Cruz (UCSC) genome browser to characterize the association of the WASp ChIP-seq-binding profile with RNA Polymerase II promoters and epigenetic marks for active and repressed transcription as well as association with peaks obtained by ChIP for specific TFs and by DamID of genome-nuclear lamina interactions for nuclear envelope lamin B1 [48] (Fig. 2a). Many of the WASp-enriched genes were associated with RNA Polymerase II-enriched genes (Fig. 2a). WASp-enriched sequences of *Tcf12* were associated with RNA Polymerase II binding and active epigenetic marks of transcription; H3K4m3, H3K9a, H3K27a, and with the epigenetic mark for active enhancers H3K4m1 (Fig. 2a and b). WASp-enriched sequences of *Vav2* and *WASf2* were associated with RNA Polymerase II binding and active epigenetic marks of transcription H3K27a and/or active enhancers H3K4m1 (Fig. 2a and b). WASp-enriched sequences were found close to the TSS of *Nfatc2*, *Trim50*, *Nebl*, *WASf2*, *Palld*, *Tcf12*, *Cct4*, *Sec24a*, *Rbm20*, *Zfp438*, *Zpbp* (Fig. 3a, Additional file 1: Figure S4A) and in deep intronic regions for *Vav2*, *Taf1b*, and other genes (Fig. 3b, Additional file 1: Figure S4B). These data suggest that WASp associates at the promoter with RNA Polymerase II and epigenetic marks for active transcription.

### Global and gene-specific transcription is repressed in WASp KO thymocytes

**Fig. 4:**
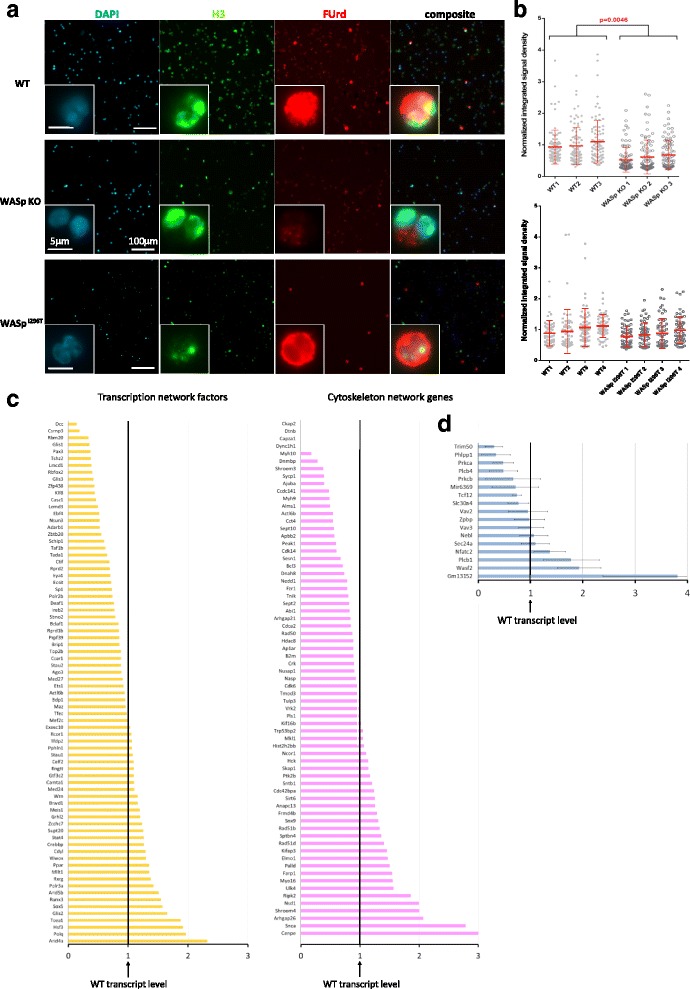
WASp functional status affects both basal and gene-specific transcription. **a** FUrd incorporation assays data for basal RNA polymerase II nascent transcripts in WT, WASp KO, and WASp^I296T^ thymocytes as determined by intensity of FUrd-rich foci (*red*). The cell nucleus is labeled with DAPI (*blue*) and histone H3 (*green*). Note that mRNA transcripts can be seen in the cytoplasm already after 15 min. **b** Statistical analysis of FUrd assay results by nested ANOVA. Each *dot* represents one image. *Top*: WT n = 3, WASp KO n = 3 in one experiment; *bottom*: WT n = 4, XLN^I296T^n = 4 in one experiment. **c**, **d** Gene expression profiling by RT-qPCR of WASp ChIP-seq genes (**c**) encoding TFs, other DNA- or RNA-binding proteins, and cytoskeletal network proteins performed on cDNA template of pooled samples: WASp KO n = 3 vs. WT n = 3 and (**d**) for common WASp-enriched genes in thymocytes and spleen CD4^+^ T cells. Expression in WASp KO thymocytes is shown and compared with expression level in WT thymocytes set to 1. Gene expression profiling by RT-qPCR was performed in three biological replicates: WASp KO n = 3 vs. WT n = 3 and in three technical replicates for each cDNA template sample with Ct variation between triplicates less than cutoff ± 0.25. Gene expression data: the mean of the technical triplicate, ΔCt, RQ value, and standard deviation between RQ of biological triplicates is indicated. See Additional file 1: Figure S5

To examine the role of WASp in global gene transcription, we performed a run-on transcription assay on living cells in which fluorine-conjugated UTP (FUrd) analogues are incorporated into nascent messenger RNA (mRNA) molecules [41, 49]. Since WASp KO thymocytes have reduced capacity to respond to T cell receptor stimulation [21, 28–30], we examined mRNA transcription in naïve thymocytes. When compared to WT thymocytes, WASp KO thymocytes showed less intense FUrd-rich foci, suggesting that WASp is required for basal transcription (Fig. 4a, b). We next compared general synthesis of nascent mRNA molecules in WT thymocytes with thymocytes expressing the constitutively active mutation of WASp, WASp^I296T^, and found similar intensity of FUrd-rich foci in WT and WASp^I296T^ thymocytes (Fig. 4a, b).

To understand how WASp influences gene transcription of specific genes, we next isolated total mRNA from WT and WASp KO thymocytes and compared gene expression using RT-qPCR. From the WASp ChIP-seq dataset of WASp-interacting genes, we selected 76 genes encoding for proteins associated with cytoskeletal networks and 76 genes encoding for proteins in transcriptional networks and performed expression profiling (Additional file 1: Figure S5). Many of these genes were differentially expressed in WT and WASp KO thymocytes (Fig. 4c), suggesting that WASp regulates gene transcription at several loci.

We next compared expression of selected genes including the 15 common WASp-interacting genes in the ChIP-seq datasets for thymocytes and spleen CD4^+^ T cells. Of these genes, 11 out of 17 genes showed lower expression in WASp KO thymocytes when compared to WT thymocytes, including *Mir6369*, *Trim50*, *Vav2*, *Phlpp1*, *Vav3*, *Prkca*, *Slc30a4*, *Plcb4*, *Prkcb*, *Zpbp*, and *Tcf12* (Fig. 4d). Six out of 17 genes showed higher expression in WASp KO thymocytes when compared to WT thymocytes, including *Sec24a*, *Nebl*, *Nfatc2*, *Plcb1*, *GM13152*, and *WASf2* (Fig. 4d). Together these data suggest that WASp is involved in both transcription activation and repression of specific genes.

### Gene regulation by nuclear WASp^I296T^

**Fig. 5:**
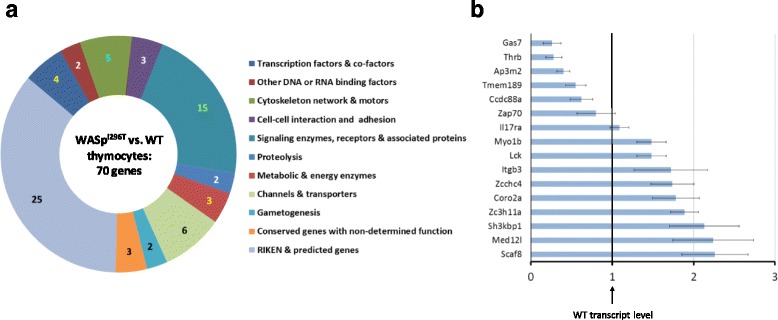
Gene regulation by nuclear WASp^I296T^. **a** Functional groups of peak-corresponding genes in WASp ChIP-seq refined datasets. **b** Gene expression profiling by RT-qPCR of WASp^I296T^ ChIP-seq genes in WASp^I296T^ vs. WT thymocytes. Expression in WASp^I296T^thymocytes is shown and compared with expression in WT thymocytes set to 1. Gene expression profiling by RT-qPCR was performed in three biological replicates: WASp^I296T^ n = 3 vs. WT n = 3 and in three technical replicates for each cDNA template sample with Ct variation between triplicates less than cut-off ± 0.25. Resulted gene expression data: the mean of the technical triplicate, ΔCt, RQ value, and standard deviation between RQ of biological triplicates is indicated. Additional file 3: sheet 3

To examine the genomic distribution of the constitutively active form of WASp [37], WASp^I296T^, we performed ChIP-seq analysis using the WASp F-8 Ab under low stringency conditions (10 mM DMA/PBS wash, Additional file 1: Figure S2D). To pre-process raw ChIP-seq data, the total number of reads were normalized and aligned against the mouse genome to identify regions enriched in WASp^I296T^ binding (Additional file 1: Figure S2F). We obtained differential binding peaks between thymocytes expressing WT WASp or WASp^I296T^ and identified 70 WASp^I296T^-enriched genes (Fig. 5a and Additional file 3: sheet 3). Functional clustering of these genes revealed that WASp^I296T^ was associated with RNA Polymerase II genes in 11 functional groups (Fig. 5a). Among the identified genes were regulators of signal transduction (Lck, ZAP70), cytoskeleton-associated proteins (Coro2a), and molecular motors (Myo1b, Supplementary file 1 sheet 3). From the ChIP-seq dataset of WASp^I296T^-interacting genes, we selected 16 genes and performed expression profiling (Fig. 5b). Of these genes, six of the 16 genes showed lower expression in WASp^I296T^ thymocytes when compared to WT thymocytes, including *Gas7*, *Thrb*, *Ap3m2*, *Tmem189*, *Ccdc88a*, and *ZAP70* (Fig. 5b). Ten of the 16 genes showed higher expression in WASp^I296T^ thymocytes when compared to WT thymocytes, including *Myo1b*, *Lck*, *Itgb3*, *Zcchc4*, *Coro2a*, *Zc3h11a*, *SH3kbp1*, *Med12I*, and *Scaf8* (Fig. 5b). Together these data suggest that thymocytes expressing constitutively active WASp^I296T^ had same intensity of basal gene transcription (Fig. 4a, b) and induced higher expression of specific genes (Fig. 5b).

### Association between WASp, TCF12, and TCF1 in the nucleus

**Fig. 6:**
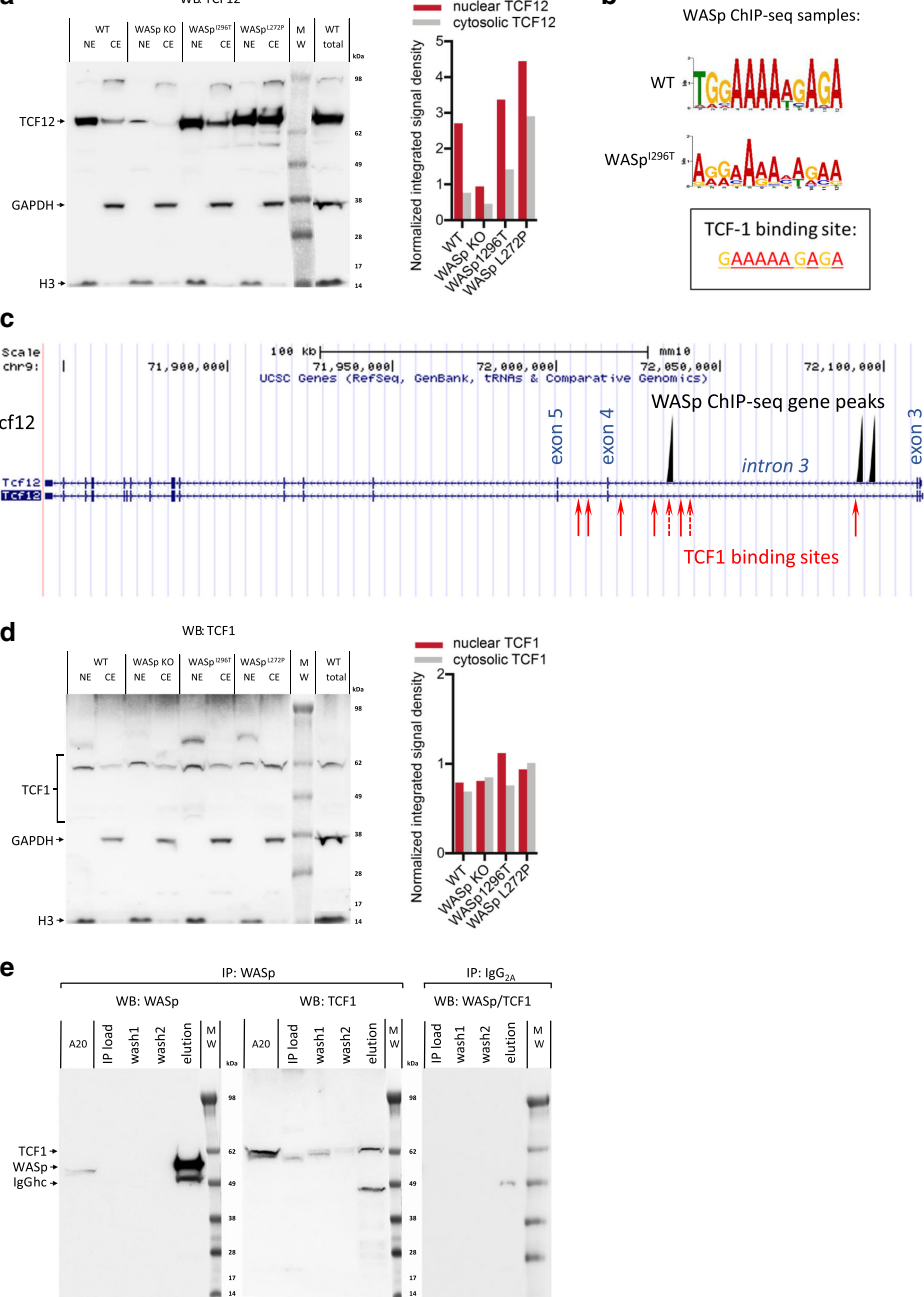
The activation status of nuclear WASp affects TCF1-mediated *Tcf12* expression. **a** TCF12 in NE and CE from WT, WASp KO, WASp^I296T^, and WASp^L272P^ thymocytes. These data were repeated twice with independent biological replicates. Detection of GAPDH was used as a sample loading control for total lysate and for cytosolic extract. Detection of histone H3 was used as a sample loading control for total lysate and for nuclear extract. The quantification of nuclear and cytosolic TCF12 in the western blot is shown in the *right panel*. **b** Consensus DNA motif determined by WASp ChIP-seq data analysis and the canonical TCF1 DNA-binding site. **c** Proximity of WASp ChIP-seq gene peak positions and TCF1-binding sites in *Tcf12* intron 3. **d** TCF1 in NE and CE from WT, WASp KO, WASp^I296T^, and WASp^L272P^ thymocytes. These data were repeated twice with independent biological replicates. Detection of GAPDH was used as a sample loading control for total lysate and for cytosolic extract. Detection of histone H3 was used as a sample loading control for total lysate and for nuclear extract. The quantification of nuclear and cytosolic TCF1 in the western blot is shown in the right panel. **e** Co-IP of WASp and TCF1 from thymocyte nuclear extracts. These data were repeated three times with independent biological replicates. Purified mouse IgG_2A_ was used as the isotype Ab-negative control IP. Heavy chain of IgG (IgGhc) is observed in eluted IP samples. Additional file 1: Figure S6 and S7 and Additional file 4

*Tcf12* was one of the 15 WASp-enriched genes present in both thymocytes and spleen CD4^+^ T cells. To understand how the activity of WASp influences expression of the *Tcf12* gene, TCF12 was examined in T cells from WT, WASp KO, WASp^L272P^, and WASp^I296T^ mice, the latter two expressing a constitutively active form of WASp [37]. Since TCF12 is mainly localized in the nucleus, we examined TCF12 in nuclear and cytosolic extracts from WT, WASp KO, WASp^L272P^, and WASp^I296T^ thymocytes. TCF12 was predominantly expressed in the nuclear extract of WT thymocytes (Fig. 6a). WASp KO thymocytes had significantly reduced TCF12 signal that was barely detected in the cytosolic extracts (Fig. 6a). This was in contrast to thymocytes expressing the constitutively active form of WASp. Both WASp^L272P^ and WASp^I296T^ thymocytes had increased TCF12 in the nuclear extract and in the cytosolic extract when compared to WT thymocytes (Fig. 6a). These data suggest that the activation status of WASp influences the level of nuclear TCF12 protein in thymocytes.

To understand how WASp regulated *Tcf12* expression, we examined if WASp contained any described DNA-binding structural motifs or documented DNA-interacting properties and found no such evidence using gene ontology tools provided by MGI at NCBI Gene resource (Additional file 4). This suggests that the association of WASp with DNA may be indirect and mediated by interaction with DNA-binding proteins. We analyzed if there was any conserved DNA-binding site(s) in the WASp-enriched sequences from the ChIP-seq datasets from WT thymocytes and from WASp^I296T^ thymocytes. Interestingly, WASp-interacting sequences in WT thymocytes were enriched for a DNA element identical to the TCF1-binding site (Fig. 6b) [47]. WASp^I296T^-interacting sequences from thymocytes were also enriched for at DNA element with similarity to the TCF1-binding site (Fig. 6b). We next examined if the *Tcf12* gene contained TCF1-binding sites. We identified several TCF1-binding sites in *Tcf12* introns 3 and 4 in close proximity to the WASp-enriched sequences (Fig. 6c, Additional file 1: Figure S6A). To examine TCF1 protein in nuclear and cytosolic extracts from WT, WASp KO, WASp^L272P^, and WASp^I296T^ thymocytes, we used an Ab that is specific for the longest TCF1 isoform that is transcriptionally active due to its binding to β-catenin [50, 51]. TCF1 was detected in all lysates with predominant localization in the nuclear extracts (Fig. 6d). To determine if WASp directly interacted with TCF1, WASp was immunoprecipitated from thymocyte nuclear extracts using the WASp F-8 Ab and found to co-immunoprecipitate with TCF1 (Fig. 6e). This suggests that WASp and TCF1 may interact to drive transcription of TCF1 pathway genes including *Tcf1* itself. In support of this, the WASp ChIP-seq dataset identified several WASp-interacting genes that are induced by TCF1 signaling (Additional file 1: Figure S6B). Together, these data suggest that nuclear WASp acts in close proximity with TCF1 and TCF12 (see tentative model in Additional file 1: Figure S7).

## Discussion

The contribution of nuclear WASp activity for T cell development remains incompletely defined. Using genome-wide analysis of the global genome occupancy of WASp in T cells, we found that WASp interacted with both intergenic and genic sequences and was enriched at TSSs of a large number of protein-coding genes. In the absence of WASp, RNA polymerase II transcription was reduced, suggesting that WASp participates in regulation of basal RNA polymerase II transcription. We identified that WASp-enriched sequences had a consensus-binding site identical to the TCF1-binding site and that WASp interacted with TCF1 in the nucleus. Moreover, we show that WASp interacted with promoter-proximal introns of the gene encoding TCF12 and that the activation status of WASp influenced the level of TCF12 protein in the nucleus.

WASp-enriched genes in thymus and spleen CD4^+^ T cell samples shared similar features in regard to functional groups and intragenic and genic occupancy. In thymocytes, we found a large fraction of WASp in the nuclear fraction when compared to the cytosolic fraction. This was in contrast to spleen CD4^+^ T cells where much less WASp was present in the nuclear fraction. This suggests that the nuclear activity of WASp is important during thymocyte development. In spleen CD4^+^ T cells, the cytosolic activity of WASp in engaging proximal T cell receptor signaling may become more important as shown in many studies [18]. We identified some common genes when comparing global gene occupancy by WASp in thymocytes and spleen CD4^+^ T cells (our datasets) with the targeted ChIP on array approach for genes that regulate Th1 and Th2 commitment [38, 39]. Among common WASp ChIP-seq enriched genes were a mouse orthologue *Mamld1* of previously reported Mastermind like1 (MAML1) as well as other genes involved in Notch signaling pathways including in thymocytes *Sbno2* and in spleen CD4^+^ T cells *cadherin-6* and *Rbpj*. We observed a number of regulatory genes associated with histone acetylation and deacetylation activities including in thymocytes *Kat6b* (histone acetyltransferase KAT6B) and in spleen CD4^+^ T cells *Kansl1* (KAT8 regulatory NSL complex subunit 1), *Sirt6* (NAD-dependent protein deacetylase sirtuin-6), and *Hdac8* (histone deacetylase 8). Previously identified genes such as *Foxp3* and *Tbx1* [38, 39] were not found in our datasets.

The signature of the WASp-enriched genes suggested that WASp serves a role both in basal RNA polymerase II transcription and in regulation of specific genes. Although basal RNA Polymerase II gene transcription was lower in WASp KO thymocytes, we identified a few exceptions where expression of specific genes was upregulated in WASp KO thymocytes. When comparing WASp-enriched genes in thymocytes and spleen CD4^+^ T cells we found 15 common genes. Several of these genes showed lower expression in WASp KO thymocytes including *Vav2* and *Vav3* that coordinate proximal T cell receptor signaling to activation of the Rho GTPases Rac1, Rac2, and Cdc42 and WASp family members. Five of the 15 common WASp-enriched genes were upregulated in WASp KO thymocytes. Of these, WASp interaction with *Nfatc2* is interesting since it suggests that nuclear WASp may regulate *Nfatc2* expression and thereby production of interleukin-2. WASp KO T cells have reduced translocation of Nfatc2 into the nucleus upon T cell receptor stimulation [30] and we show here that this may be compensated by increased expression of *Nfatc2* in WASp KO thymocytes. *WASf2*, that encodes for WASp family member WAVE2 also showed increased expression in WASp KO thymocytes, suggesting that loss of WASp stimulated transcription of WAVE2. The WASp family members are known to be able to compensate for each other to support actin polymerization. N-WASp can compensate for loss of WASp in development and function of B and T cells [21, 52–54]. However, the compensation is not complete as evident in increased frequency of autoreactive B and T cells that only express N-WASp [20, 53–55]. Our data provide an example where this cross-compensation between WASp family members may occur in the nucleus through interaction of WASp with *WASf2* and increased gene expression of *WASf2* in WASp KO cells.

Several WASp family members are present in the nucleus and it is feasible to assume, based on their activity in the cytoplasm, that they at least in part may exert function in the open conformation exposing their VCA domain. We provide evidence for that the active open conformation of WASp in WASp^L272P^ and WASp^I296T^ thymocytes showed same intensity of basal gene transcription when compared to WT thymocytes, and had higher expression of specific genes. Moreover, WASp^L272P^ and WASp^I296T^ thymocytes had more nuclear TCF12 protein when compared to WT thymocytes, perhaps indicating that a fraction of WT WASp resides in an autoinhibited conformation that prevents over-activation of WASp. Previous data suggest that gene transcription regulated by a WASp family member can be both dependent and independent on the VCA domain. In human T cells differentiated into Th1 cells, the WASp-VCA domain is dispensable for gene transcription of Tbx12-target genes [56]. Instead, sumoylation of WASp at lysine residues is important for transcription of this set of genes [57]. In Xenopus oocytes, WAVE1-induced gene transcription and reprogramming is independent of the WAVE1 VCA-like domain [58]. There are also examples of WASp family members that require the VCA domain and actin for gene transcription. In HeLa cell nuclei, N-WASP is part of a large nuclear–protein complex containing polypyrimidine-tract-binding-protein-associated splicing factor–non-Pou-domain octamer-binding protein/p54nrb (PSF–NonO), nuclear actin, and RNA polymerase II [59]. The PSF–NonO complex is involved in the regulation of many cellular processes, such as transcription, RNA processing, DNA unwinding, and repair. Interestingly, an N-WASPΔVCA mutant localizes within the nucleus but is defective in actin polymerization resulting in significantly decreased gene transcription [59]. WASH is located in the nucleus of hematopoietic stem cells where it associates with the nucleosome remodeling factor (NURF) complex and assists the NURF complex to the promoter of the *c-Myc* gene necessary for hematopoietic stem cell differentiation [60]. The nuclear activity of WASH is dependent on nuclear actin polymerization induced by the WASH VCA domain [60]. Together, these studies raise the possibility that the multiple domains of WASp family members may have unique biological functions in the nucleus and that nuclear activity is regulated by the VCA domain as well as other functional domains similar to the role of WASp family members in the cytoplasm. In vitro, dimers or oligomers of WASp family VCA domains bind with 100-fold higher affinity to Arp2/3 when compared to monomers of VCA domains [61]. This suggests that homo- and hetero-dimerization of active WASp provides an additional layer of regulation beyond conformation and functional domains. Our data lend evidence to the open conformation of WASp being required for global gene occupancy and that constitutively active WASp in WASp^L272P^ and WASp^I296T^ thymocytes induced increased transcription of specific genes including *Tcf12*.

Here we identify that WASp targeted genes were enriched for a DNA-binding site identical to the TCF1-binding site. TCF1 is one of the earliest transcriptional regulators induced in thymus and essential for T cell commitment [62–64]. TCF1 is most abundant in CD4^+^CD8^+^ double-positive thymocytes and its absence compromises CD4^+^CD8^+^ thymocyte survival [65, 66]. TCF1 constitutively interacts with DNA and mediates repression of gene transcription when bound to Groucho and activation of transcription when bound to β-catenin [67–69]. Bioinformatic analysis suggests that WASp lacked DNA-binding properties. Instead, WASp interacted with TCF1 leading to transcription of a subset of target genes downstream of TCF1. This indicates that WASp in complex with TCF1 may regulate transcription of a subset of some but not all TCF1 pathway genes. This suggests that the WASp-TCF1 interaction may be complex and modulated by other binding partners and/or modulators of transcription. Deletion of TCF1 specifically in CD4^+^CD8^+^ double-positive thymocytes leads to a skewed CD4/CD8 ratio with increased number of CD8^+^ T cells [70]. Despite the reduction in basal RNA Polymerase II transcription and TCF1 pathway genes such as *Tcf12*, WASp KO thymocytes develop normally in regard to number of CD4^+^ and CD8^+^ single-positive thymocytes [21]. However, WASp KO mice have decreased CD4/CD8 T cell ratio in peripheral organs, leading to increased accumulation of CD8^+^ T cells in skin, spleen, and lymph nodes [71]. Moreover, WASp-deficient thymocytes and T cells have reduced capacity to respond to T cell receptor stimulation [21, 28–30]. Therefore, it is not surprising that an increasing number of reports have identified aberrations in T cell development and differentiation in WASp deficiency including an oligoclonal T cell receptor repertoire often found in autoreactive T cells [23–27], increased number of CD44^+^CD62L^–^ effector memory T cells [21, 71], and T cell-driven autoimmune colitis [20–22, 72]. Early studies of postmortem examination of WAS patients showed thymic hypoplasia [73], suggesting reduced thymic function and T cell development. A reduction in peripheral blood T cell number is apparent already at an early age in WAS patient and older patients have marked T cell lymphopenia [74–76]. Together, this suggests that WASp deficiency leads to aberrant T cell development and that thymocytes have reduced capacity to undergo central tolerance checkpoint to delete autoreactive T cells. The data presented here support this idea by showing that the nuclear activity of WASp is important to regulate gene transcription in a transcriptional network with TCF1 and TCF12 during T cell development. Constitutive activation of the β-catenin–TCF1 pathway is associated with genomic instability and T cell lymphomas [77, 78]. Future studies of nuclear WASp may reveal whether dysregulation of β-catenin–TCF1 signaling is associated with increased frequency of lymphoma in WAS patients.

## Conclusions

To identify the global genome occupancy of WASp, we have used ChIP-seq for WASp in thymocytes and spleen CD4^+^ T cells. We found that WASp interacted with coding and non-coding DNA regions and that WASp deficiency resulted in decreased RNA polymerase II transcription. Finally, we identified a molecular network important for T cell development placing nuclear WASp in close proximity with TCF1 and TCF12.

## Additional files

Additional file 1: Supplementary Figures S1–S7. (PDF 1766 kb)

Additional file 2: Primer list. (XLSX 21 kb)

Additional file 3: WASp ChIP-seq data. (XLSX 53 kb)

Additional file 4: DNA-binding proteins by GO. (XLSX 313 kb)

## Abbreviations

Ab: Antibody
Arp: Actin-related protein
ChIP-seq: Chromatin immunoprecipitation and deep sequencing
FUrd: Fluorine-conjugated UTP
HEB: HeLa E-box-binding protein
IP: Immunoprecipitation
JMY: Junction-mediating and regulatory protein
Lef: Lymphoid enhancer factor
Nfatc: Nuclear factor of activated T cells
SCAR: Suppressor of the cyclic AMP receptor
SWI/SNF: SWItch/Sucrose Non-Fermentable complexes
TCF: T cell factor
TCR: T cell receptor
Th: T helper
TSS: Transcriptional start site
VCA: Verprolin cofilin acidic
WASH: WASp and SCAR homologue
WASp: Wiskott–Aldrich syndrome protein
WAVE: WASp-family verprolin-homologous protein
WT: Wild type
XLN: X-linked neutropenia
