# Supplementary material for: Nuclear Wiskott–Aldrich syndrome protein co-regulates T cell factor 1-mediated transcription in T cells
Source: Genome Med. 2017 Oct 27;9:91. doi: 10.1186/s13073-017-0481-6 (PMC5660450; doi:10.1186/s13073-017-0481-6)

Figure S1

Quality control of spleen CD4+ T cells isolation

FACS data shows 94-96% purity of spleen $CD4^{+}CD3^{+}$ T cells used for ChIP-Seq analysis.

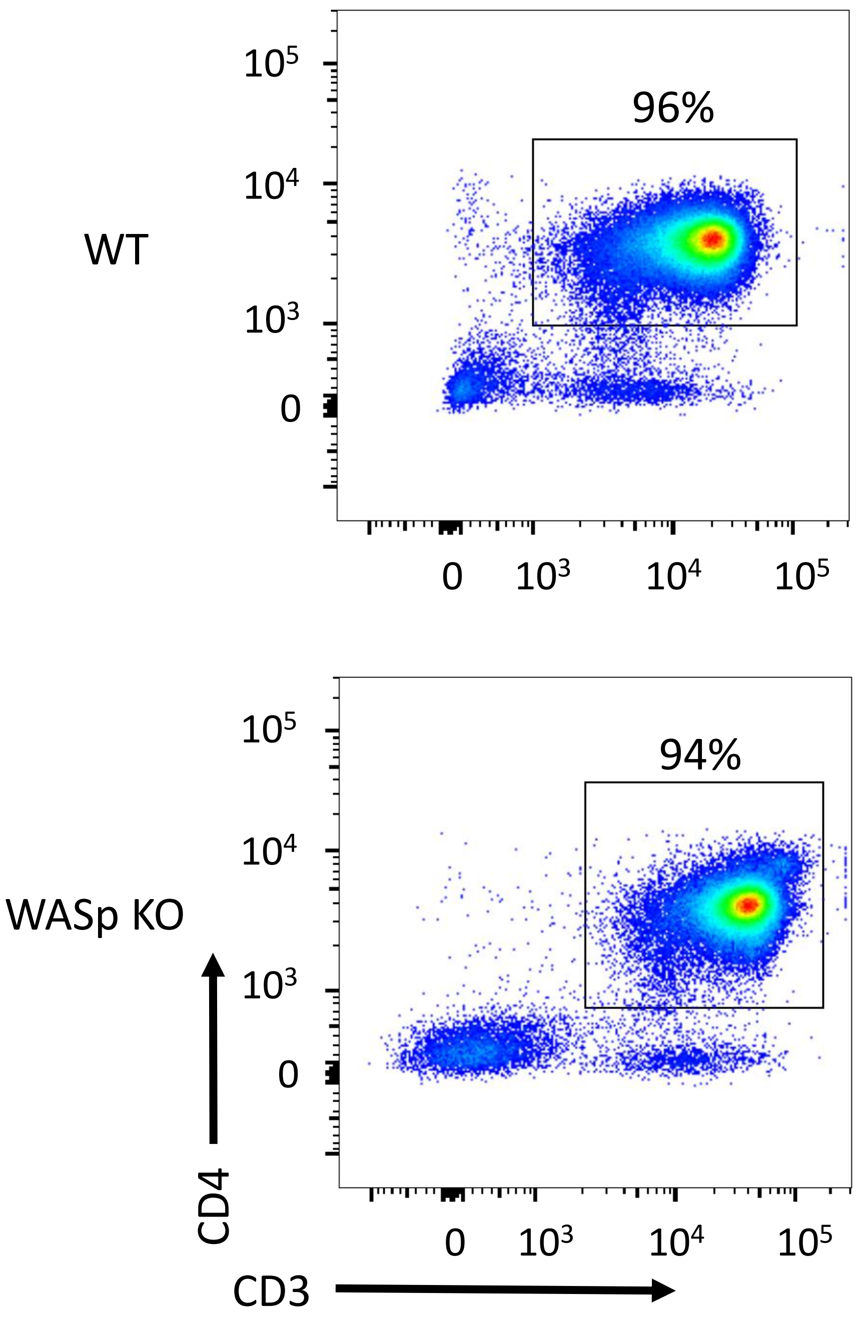

Figure S2

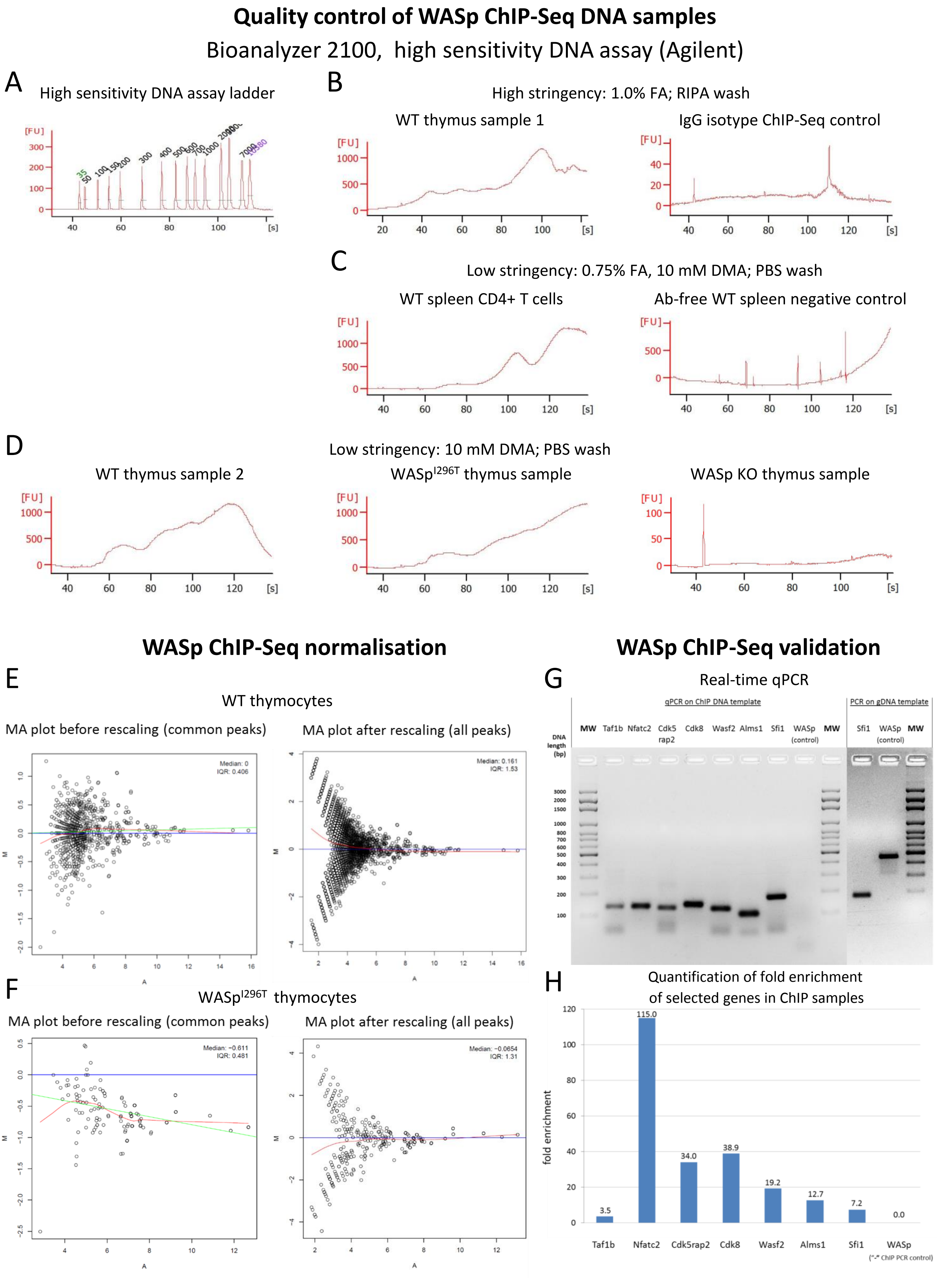
Quality control of WASp ChIP-Seq DNA samples
Bioanalyzer 2100, high sensitivity DNA assay (Agilent)
A
High sensitivity DNA assay ladder
B
High stringency: 1.0% FA; RIPA wash
WT thymus sample 1
IgG isotype ChIP-Seq control
C
Low stringency: 0.75% FA, 10 mM DMA; PBS wash
WT spleen CD4+ T cells
Ab-free WT spleen negative control
D
Low stringency: 10 mM DMA; PBS wash
WT thymus sample 2
WASp^{I296T} thymus sample
WASp KO thymus sample
[FU]
[s]
WASp ChIP-Seq normalisation
E
WT thymocytes
MA plot before rescaling (common peaks)
MA plot after rescaling (all peaks)
Median: 0
IQR: 0.406
Median: 0.161
IQR: 1.53
F
WASp^{I296T} thymocytes
MA plot before rescaling (common peaks)
MA plot after rescaling (all peaks)
Median: −0.611
IQR: 0.481
Median: −0.0654
IQR: 1.31
M
A
WASp ChIP-Seq validation
G
Real-time qPCR
qPCR on ChIP DNA template
PCR on gDNA template
DNA length (bp)
MW
Taf1b
Nfatc2
Cdk5 rap2
Cdk8
Wasf2
Alms1
Sfi1
WASp (control)
H
Quantification of fold enrichment of selected genes in ChIP samples
fold enrichment
3.5
115.0
34.0
38.9
19.2
12.7
7.2
0.0
Taf1b
Nfatc2
Cdk5rap2
Cdk8
Wasf2
Alms1
Sfi1
WASp
("-" ChIP PCR control)

Figure S3

Analysis of input controls from thymocyte and spleen CD4$^+$ T cell ChIP samples

show more than a 10-fold enrichment of reads in thymocyte (A) and spleen CD4$^+$ T cell (B) WASp ChIP-Seq. Mean of fold enrichment and standard deviation (SD) are indicated (red).

A

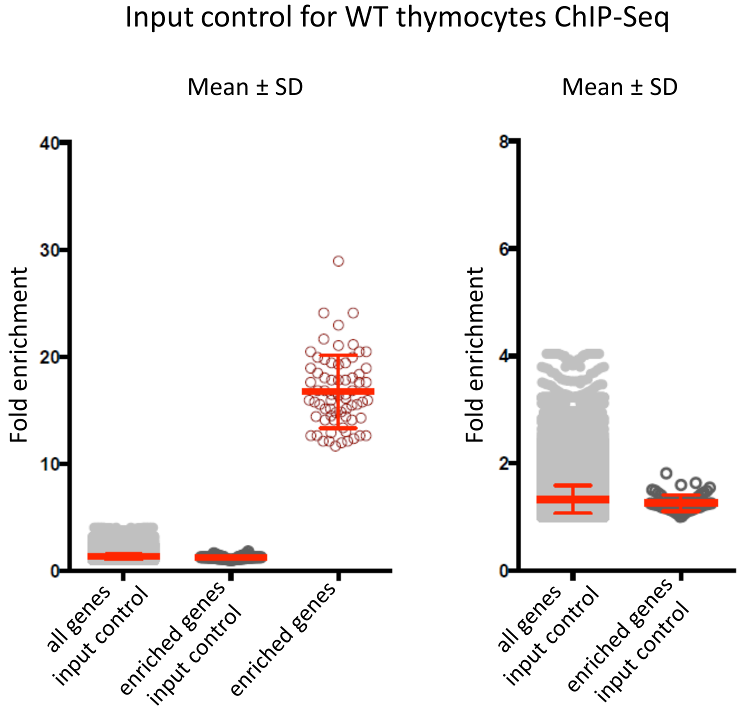

B

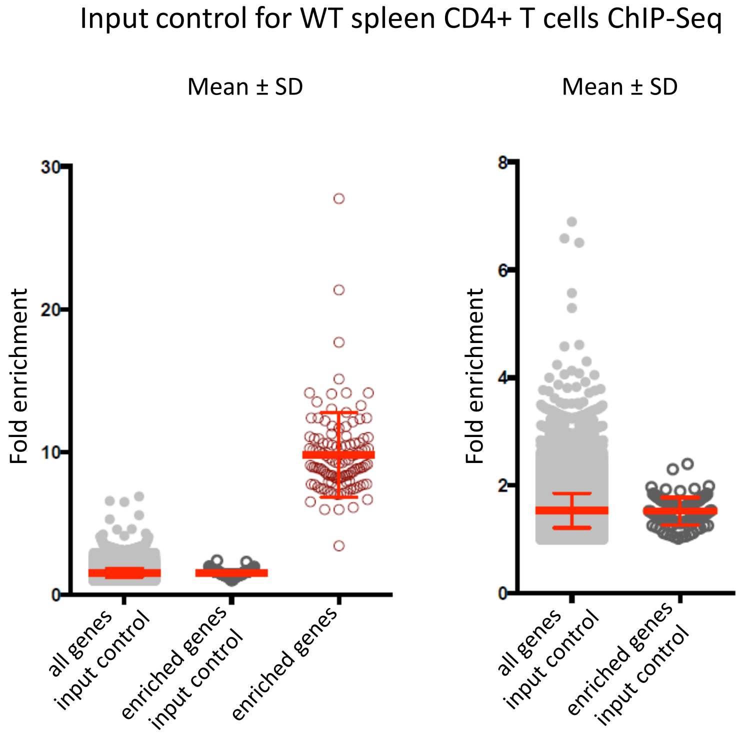

Figure S4

Alignment of selected WASp ChIP-Seq gene peaks

(A) Selected genes with shorter distance range (0.2-5 kb) between WASp ChIP-Seq gene peak position and nearest exon.

(B) Selected genes with longer distance range (5-50 kb) between WASp ChIP-Seq gene peak position and nearest exon.

Note that *Nfatc, Trim50, Vav2, Nebl, WASf2, Taf1b*, and *Tcf12* are also shown in Figure 3.

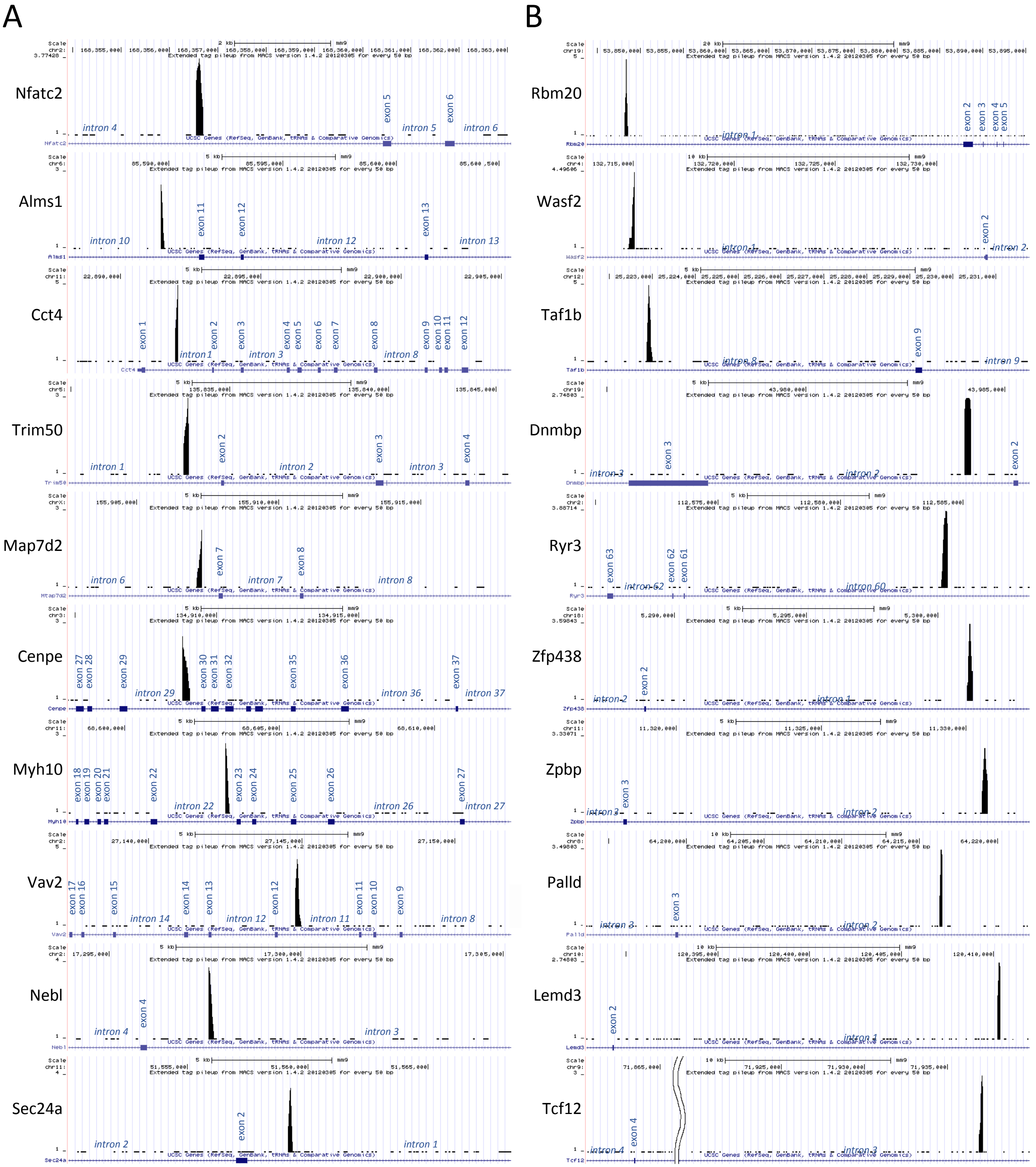

Figure S5

ChIP-Seq genes validation by RT-qPCR in WASp KO lymphocytes

The percentage of validated genes within the selected groups is indicated.

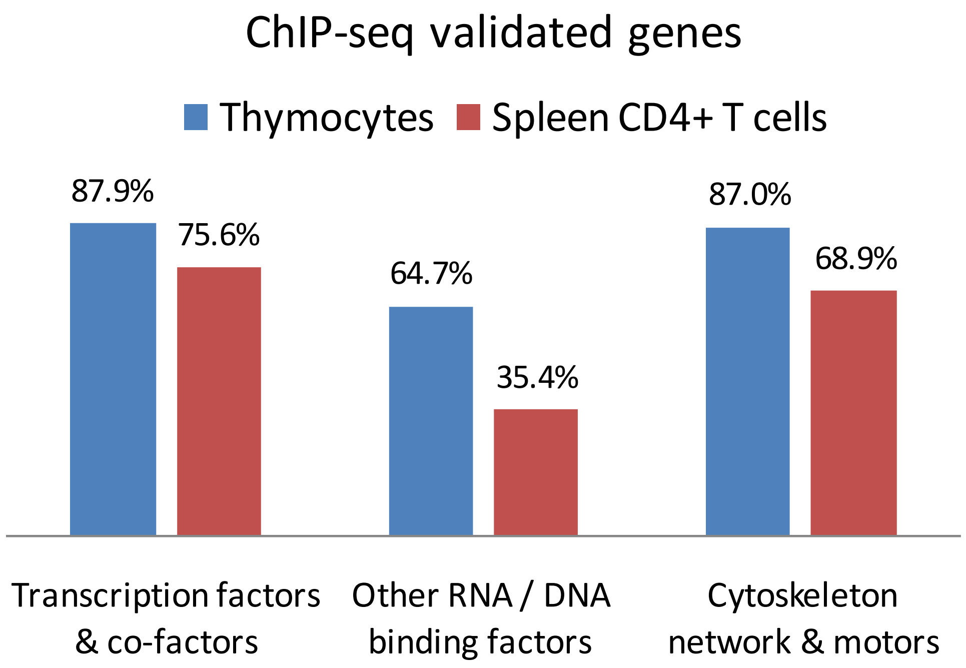

Figure S6

WASp acts in proximity with TCF1

A

An example for one of several TCF1 binding sites found in *Tcf12* intron 3

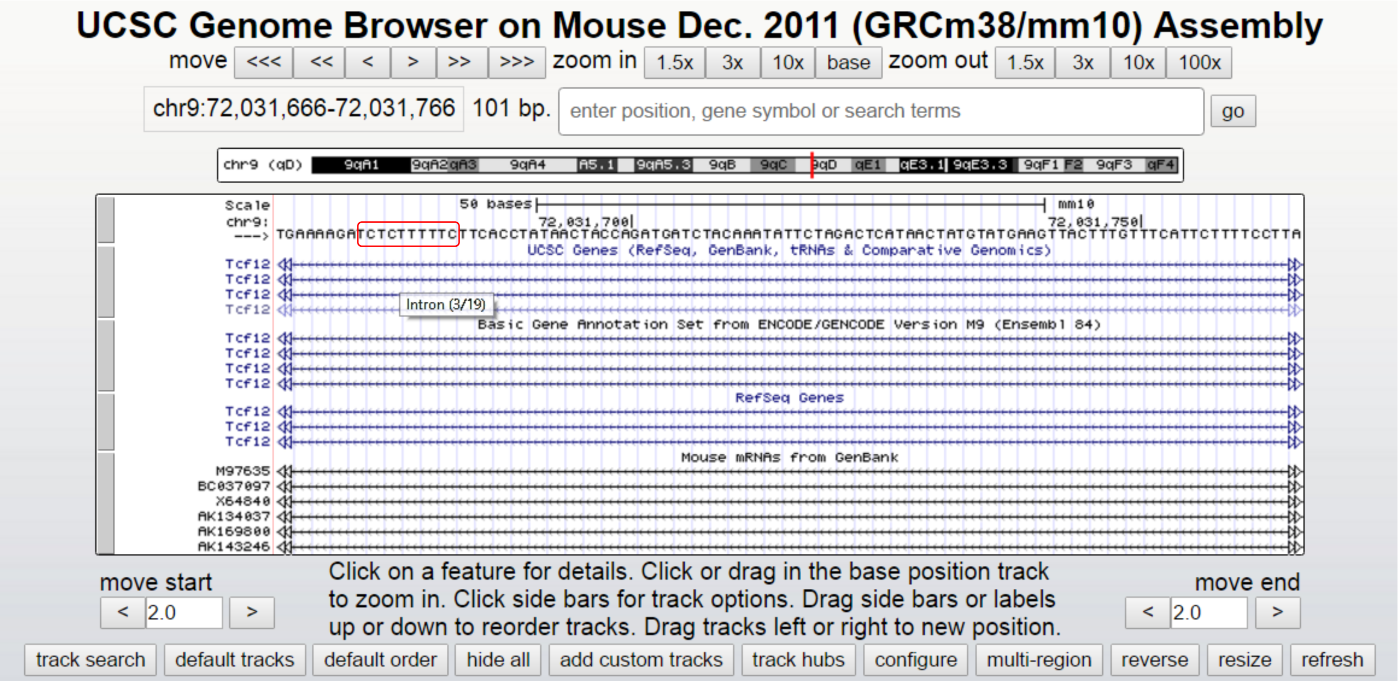

B

A subset of TCF1 pathway genes were identified in the WASp ChIP-Seq data set

Gene id	Symbol	Gene name	Source
22413	Wnt2	wingless-type MMTV integration site family, member 2	thymocytes
21414	Tcf1	transcription factor 1	thymocytes
21406	Tcf12	transcription factor 12	thymocytes
235542	Ppp2r3a	protein phosphatase 2, regulatory subunit B", alpha	thymocytes
329650	Med12l	mediator complex subunit 12-like	thymocytes
14812	Grin2b	glutamate receptor ionotropic, NMDA 2B	spleen CD4+ T cells
80707	Wwox	WW domain-containing oxidoreductase	spleen CD4+ T cells
56297	Arl6	ADP-ribosylation factor-like protein 6	spleen CD4+ T cells
108909	Aida	axin interactor, dorsalization associated	spleen CD4+ T cells
665113	Tnik	traf2 and NCK-interacting protein kinase	spleen CD4+ T cells
18647	Cdk14	cyclin-dependent kinase 14	spleen CD4+ T cells
407821	Znrf3	E3 ubiquitin-protein ligase ZNRF3; zinc/RING finger protein 3	spleen CD4+ T cells
14734	Gpc3	glypican-3	spleen CD4+ T cells
102334	Ankrd10	ankyrin repeat domain-containing protein 10	spleen CD4+ T cells

Figure S7

WASp -TCF1 RNA Pol II transcription co-regulation model

A schematic model for how recruitment of WASp and the RNA Polymerase II transcription machinery may regulate gene expression downstream of the TCF1 pathway. Indicated is a subset of TCF1 pathway genes identified in the WASp ChIP-Seq data set.

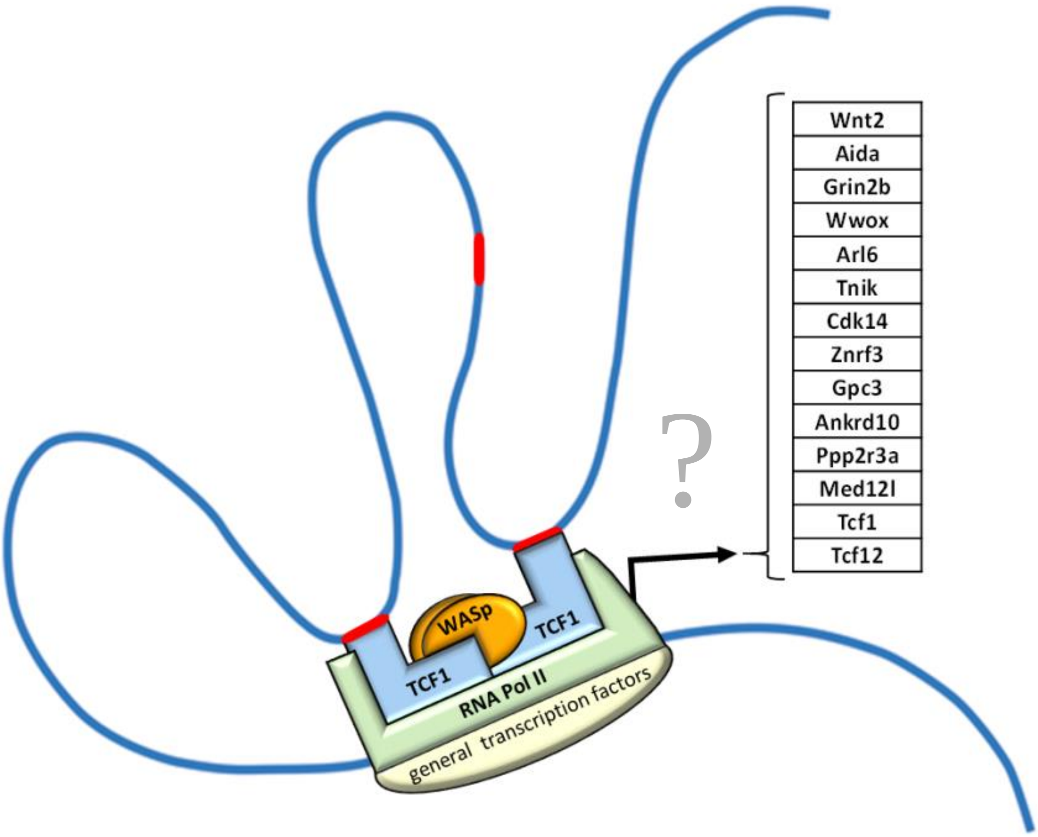

Supplement: Supplementary file 1 — Supplementary Figures S1–S7. (PDF 1766 kb) [file 13073_2017_481_MOESM1_ESM.pdf]
